# Impact of Chronic Statins Use on the Development of Esophagitis in Patients with Gastroesophageal Reflux Disease

**DOI:** 10.1155/2019/6415757

**Published:** 2019-02-03

**Authors:** Tawfik Khoury, Amir Mari, Hana Amara, Mohamed Jabaren, Abdulla Watad, Wiliam Nseir, Wisam Sbeit, Mahmud Mahamid

**Affiliations:** ^1^Gastroenterology and Endoscopy United, The Nazareth Hospital, EMMS, Nazareth, Bar Ilan University, Tel-Aviv, Israel; ^2^Institute of Gastroenterology and Liver Disease, Galilee Medical Center, Bar Ilan Faculty of Medicine, Safed, Israel; ^3^Cardiology Department., Haemek Medical Center, Afula, Israel; ^4^Department of Medicine ‘B' Sheba Medical Center, Tel-Hashomer, Israel; ^5^Internal Medicine Department A, Badeh Barouch Medical Center, Poria, Israel

## Abstract

**Background and Aims:**

We aimed to assess whether chronic statins used (> 6 months) were protective of the development of esophagitis in patients with gastroesophageal reflux disease. In the presence of esophagitis, complications such as strictures, Barrett's esophagus, and adenocarcinoma were the most common. Statins, lipid lowering drugs with a pleiotropic effect, are recently implicated in various pathologies. Nevertheless, the possible impact of statins in esophagitis development has never been assessed.

**Methods:**

We performed a retrospective, cross-sectional, single center study that included 4148 gastroesophageal reflux disease patients from 2014 and 2018 at EMMS Nazareth Hospital. We divided the patients into 5 groups. The groups were split into positive control group, which was the nonesophagitis group, and the other 4 groups were A-D (as per Los Angeles classification).

**Results:**

Overall, out of the 4148 patients included, 48% were males and 2840 patients were in the control group. In groups A, B, C, and D there were 818, 402, 72, and 16 patients, respectively. Logistic regression analysis revealed that chronic statins usage is protective by preventing development esophagitis (OR 0.463 [95%CI 0.370–0.579], p < 0.0001). NSAIDS use, Hiatus hernia, and* H*.* pylori* were promoting factors (OR, 1.362, 1.779, and 1.811; 95% CI, 1.183-1.569, 1.551-2.040, and 1.428-2.298; P<0.0001, P<0.0001, and P<0.0001, respectively).

**Conclusion:**

Using chronic statins was protective to the development of esophagitis among GERD patients. Our findings of potential clinical application mandate further randomized controlled trials to better assess the impact of statins on esophagitis.

## 1. Introduction

Gastroesophageal reflux disease (GERD) is chronic condition characterized by exacerbation and remission pattern [1, 2]. Recently, there have been reports of an increase in the incidence of GERD, accompanied with increment in the incidence of reflux esophagitis [3]. However, only two-thirds of GERD patients ultimately develop esophagitis. Reported risk factors, for the development of reflux esophagitis in the setting of GERD, include long standing reflux disease, smoking, higher body mass index, and male gender [4, 5].

As of today, endoscopy is the only method for diagnosis of reflux esophagitis and for grading its severity. The spectrum of endoscopic findings varies and may range from nonerosive reflux disease (NERD) which is the most common endoscopic presentation of GERD to erosive esophagitis. The latter entity is further segmented, according to the severity of esophageal mucosal damage, and ranges from minimal mucosal changes such as breaks passing through erosions, ulcers, stricture formation, and malignant changes. Previous studies have shown that the risk for complications and poor prognosis in GERD patients depends on the endoscopic mucosal findings observed at the initial GERD diagnosis [6, 7].

Continuingly, reflux esophagitis severity has been consistently evaluated and reported by the most commonly used classification system called Los Angeles scale [8, 9] which classifies esophagitis stages from A to D, with D being the most severe disease [8]. Yet, proton pump inhibitors (PPI) are the most effective treatment for GERD patients. The most favorable results of PPI have been consistently shown in numerous clinical trials (70% if esophagitis exist, 50% if NERD, and 30 % in cases of functional heartburn) [10, 11]. In addition, only a few pharmacotherapies are currently under consideration for the treatment of GERD and reducing the transient lower esophageal sphincter relaxation rate, decreasing esophageal sensitivity, and enhancing esophageal motility. However, the present data is still unambiguous and further studies are needed to establish their efficacy [12].

Statins (3-hydroxy-3-methylglutaryl-coenzyme A (HMG-CoA) reductase inhibitors) primarily used as lipid lowering agents, have recently been identified to have various beneficial effects, including anti-inflammatory, anticoagulant, antiviral, antioxidative, antineoplastic, and improving endothelial functions [13–15]. The anti-inflammatory and immunomodulatory roles of statins may be explained by inhibiting IFN-*γ*-pathway-macrophage MHC class II expression and could modulate T-cell activation through the effects of LFA-1/ICAM-1 [16, 17].

While statins are mainly implicated in the treatment of hyperlipidemia and cardiovascular diseases, they have been lately shown to have the potential to protect against other disease states. This includes cancerous diseases [18], Alzheimer, dementia, osteoporosis, vitiligo, fatty liver, rheumatoid arthritis, and other liver autoimmune and viral infectious diseases [19]. Given its pleiotropic effects, we aimed to investigate whether the use of chronic statin impacts the development and the severity grade of esophagitis in GERD patients.

## 2. Methods and Materials

We conducted a retrospective, case control study between 2014 and 2018 at EMMS Nazareth Hospital, in the north of Israel. The study population is comprised of male and female patients who underwent gastroscopy due to atypical GERD symptoms, alarm symptoms of the upper gastrointestinal tract, refractory GERD symptoms, and nonresponders to antiacids. Typical GERD symptoms, according to the recommendation guideline released from the American College of Gastroenterology [20], are defined as heartburn and regurgitation symptoms. The patient inclusion criteria included those who display upper gastroscopy (performed due to atypical symptoms, alarm symptoms, refractory GERD, and nonresponders) in order to assess the degree of reflux esophagitis as evaluated by Los Angeles criteria [8, 9] and to rule out other causes. The current study received ethical approval from the hospital ethical committee and was conducted according to the Helsinki guidelines. Informed consent was waived due to the noninterventional retrospective nature of the study.

### 2.1. Data Collection

All data (demographics, clinical parameters, and endoscopic findings) were analyzed and retrieved from the central hospital archive section and electronic reports, including the endoscopy reports with pictures. Our senior gastroenterologist had reviewed all gastroscopy electronic reports and assessed the presence of esophagitis, its related degree of severity, and the presence of any other GERD-related complications, such a peptic stricture, Barrett's esophagus, and adenocarcinoma. The cohort patients were categorized according to the degree of esophagitis as per the Los Angeles classification.

### 2.2. Study Endpoints

The primary endpoint of the study was to assess whether the usage of chronic statins (20-40 mg per day for ≥ 6 months) was protective to the development of esophagitis in GERD patients. Secondary endpoint was to assess whether other factors that are found were protective to the development of esophagitis and to assess whether chronic statin was protective from Barrett's esophagus and adenocarcinoma. The univariate and the multivariate regression analysis comparisons were done between the GERD none-esophagitis group versus all grades of esophagitis (from A to D) and between the advanced esophagitis grades (C and D) as compared to initial esophagitis grades (A and B). Furthermore, the baseline and demographic characteristics were reported for each esophagitis group separately, as proposed above by Los Angeles classification. The positive control group included patients with GERD without esophagitis. Group A included patients with esophagitis grade A. Group B included patients with esophagitis grade B. Group C included patients with esophagitis grade C, and group D included patients with esophagitis grade D.

### 2.3. Statistical Analysis

Characteristics of participants were presented with descriptive statistics as arithmetic means (±SD) or range for continuous variables, or as frequencies (percentages) for categorical variables. The association between statins use and the risk of development of esophagitis was assessed with univariate tests (chi-squared for categorical variables). To measure the association between statins exposure and the risk of esophagitis, we used logistic regression analysis reporting odds ratio and confidence intervals. Figures with p-value less than 0.05 were considered statistically significant. Statistical analyses were carried out with commercial software, Statistical Package for Social Science (SPSS version 24.0, IBM, Chicago, IL, USA).

## 3. Results

### 3.1. Demographics and Baseline Characteristics

Overall, we included 4148 patients with clinical diagnosis of GERD based on typical symptoms of heartburn and regurgitation. In the control group, we included 2840 patients. In groups A, B, C, and D there were 818, 402, 72, and 16 patients, respectively. The study cohort consisted of 48% males and 52% females. The average age for the positive control group and the esophagitis groups, A, B, C, and D, was 50.2 was 46.9, 47.9, 53, and 65 years, respectively. Notably, the average age for the advanced esophagitis grades (groups C and D) was significantly higher than the earlier grades (55.2 versus 47.2 years, respectively, P<0.0001). Forty-seven percent in the positive control group were smokers as compared to 77% in the esophagitis groups (P<0.0001). Additionally, in the advanced esophagitis groups (C and D), 86 % were smokers as compared to the early esophagitis grades (A and B), P=0.02. Furthermore, 3.7% in the positive control consumed alcohol as compared to 4% in the esophagitis groups (P=0.3). Similarly, there was no difference in alcohol consumption between the advanced esophagitis grades as compared to the initial stages (6.8% versus 4%, respectively, P=0.08). However, when analyzing each grade of esophagitis alone, we found that 18.7% of patients with grade D esophagitis (group D) were consumers of alcohol as compared to the other groups (P<0.01) (Table 1). Moreover, only 8 (0.2%) patients were diagnosed with nondysplastic Barrett's esophagus, while no patients were diagnosed with esophageal carcinoma. All patients with Barrett's were recruited to periodic surveillance program as recommended by professional guidelines.

### 3.2. Protective Factors from the Development of Esophagitis

Several factors were examined to assess the protective potential on the development of esophagitis. Chronic use of statins was significantly associated with reduced incidences and milder degrees of esophagitis. In the positive control group, 15.8% of patients were chronic statin consumers as compared to 8% of the esophagitis groups (P<0.0001). Notably, the protective effect of statins was most prominent when comparing the control group to groups A to B (P<0.0001) and showed an inclination for protection when comparing the control group to group c (P=0.07). Moreover, there was no significant difference in statin use between the advance esophagitis grades as compared to the initial grades (9% versus 7.9%, P=0.3) (Table 2). Logistic regression analysis revealed that chronic statins use is protective in preventing esophagitis when compared to GERD patients without esophagitis to patients with esophagitis ((OR 0.463 [95%CI 0.370–0.579], p < 0.0001).

We conducted further subanalysis when comparing each esophagitis group with the positive control group by logistic regression analysis and identified the OR for the control group as compared to groups A, B, C, and D to be 0.497 [95%CI 0.381–0.647], p < 0.0001), 0.366 [95%CI 0.243–0.551], p < 0.0001), 0.036 [95%CI 0.002–0.583], p=0.01), and 0.354 [95%CI 0.046–2.687], p=0.3), respectively (Table 3). There was no association between chronic statins use and the risk of Barrett's esophagus and esophageal cancer (P=0.47). No other significant predictors could be identified to reduce the risk of esophagitis development.

### 3.3. Factors Associated with Increased Risk of Esophagitis

Factors that were associated with increased risk of esophagitis, including the presence of* Helicobacter pylori* infection, were showed in 66% of patients in the positive control group, as they had a positive Hp as compared to 70% in the esophagitis groups (P=0.02). On subgroups analysis, the prevalence of Hp was in 70%, 68%, 79%, and 88% in groups A, B, C, and D, respectively. Logistic regression analysis revealed the OR of Hp infection in the esophagitis groups to be 1.811 [95%CI 1.428–2.298], p<0.0001). Similarly, the presence of hiatal hernia was associated with increased risk of esophagitis (28.5% in the control group as compared to 41.4% in the esophagitis groups, P=0.001), with OR of 1.779 [95%CI 1.551–2.040], p<0.0001). Other factors associated with increased risk of esophagitis were nonsteroidal anti-inflammatory use (P<0.0001) with OR of 1.362 [95%CI 1.183–1.569], p<0.0001) (Tables 2 and 3).

## 4. Discussion

The prevalence of GERD in the western countries is estimated to be of 8%–33% and involves all age groups as it is associated with massive economic burdens and health resources consumption, mainly due to medication prescriptions and diagnostic procedures [21, 22]. Esophagitis phenotype of GERD is associated with more mucosal complications, Barret's esophagus, and adenocarcinoma; hence it is vital to maintain control of it. Statins are drugs originally designed to reduce blood cholesterol and are used widely with high safety and efficiency to reduce cardiovascular morbidity and mortality [23]. In the last few decades, statins are being reexplored and considered to have pleiotropic effects. The anti-inflammatory effects of statins have been observed in the PRINCE study, by Michelle et al. Pravastatin has been shown to reduce CRP after 12 and 24 months of treatment, regardless of lipid profile baseline manner levels among patients with atherosclerosis, emphasizing its anti-inflammatory role within the atherosclerotic plaques [24].

Experimental studies in cell cultures and animal models revealed that statins can affect inflammatory factors and pathways such as inhibiting the expression of adhesion molecules and chemokines that recruit inflammatory cells, resulting in net anti-inflammatory effects [25]. Only a few studies have aimed at investigating the role of statins in various upper gastrointestinal symptoms. Fujii et al. assessed the possible association between statins and upper gastrointestinal disorders such as peptic ulcer disease and erosive esophagitis. The authors examined 120 gastroduodenal ulcer cases and 146 reflux esophagitis cases. Their results revealed that statin use did not increase the risk of peptic ulcer (OR 1.2; 95% CI 0.7-2.1), while in patients with reflux esophagitis, statins use might be protective (OR 0.8; 95%CI 0.5-1.4) [26]. Our study showed similar results with more powerful protective effects (OR 0.463).

Additionally, we have noticed that statins use was associated with milder degrees of esophagitis; one might speculate that statins use is protective mainly in the initial process of inflammation. Therefore, this may suggest that statins have a protective role in esophagitis development in GERD patients but not in preventing progression of esophagitis. This may be explained, at least partially, by a rheumatoid arthritis mice model treated with statins. Fuji et al. showed that statins attenuated several pathways associated with inflammation onset, such as expansion of Th1 cells, which at least in part drive the production of proinflammatory cytokines by macrophages [27]. This important finding is poorly understood; however, it may indicate that, in the advanced esophagitis stages, the fibrotic component dominates over the inflammatory component, thus leading to reduced response to statins. This observation should be further investigated using cell culture, animal model, and clinical randomized controlled trails to better define the protective role of statins in esophagitis development.

The female predominance of our overall cohort is of interest since male sex was traditionally reported as the predominant one; however when inspecting the subgroups, male predominance was observed within the more advanced esophagitis groups and female sex was associated with NERD. Our findings were compatible with the current knowledge and literature available today [28]. In addition, patients within the advanced stages of esophagitis were older. This is in keeping with current knowledge available and may be hinged to weaken lower esophageal sphincter among the elderly, ineffective esophageal peristalsis, inadequate sensation, reporting of reflux symptoms, and polypharmacy [28].

Most studies showed that NERD phenotype is more common than esophagitis phenotype (generally more than the half of patients with GERD, present as NERD phenotype) [29]. This data corresponds with our cohort's findings as presented. Past studies regarding the possible association between cigarette smoking and GERD have revealed conflicting results [30, 31]. Laboratory studies have shown an inhibitory effect of cigarettes to the lower esophageal sphincter [32, 33]. We found active smoking to be associated with esophagitis when compared to the control group. Nonetheless, no significant differences observed between the different esophagitis severity groups. Alcohol consumption and the presence of a hiatus hernia are also well-known risk factors for GERD and esophagitis [34]. The presence and size of a hiatal hernia are associated with an incompetent LES, ineffective esophageal motility, more severe mucosal damage, and prolonged acid exposure time [35].

In our study, Hiatal hernia and alcohol consumption were more prevalent among patients within the esophagitis group, compared to the control, and alcohol consumption was significantly associated with the most sever esophagitis subgroup, D. These findings are in agreement with the current knowledge of the impact of both variables on esophagitis [34, 35].

Expectedly, chronic use of NSAIDS was associated with esophagitis and shown to be a promoting factor in regression analysis. NSAIDS is known risk factors for esophagitis development as shown previously and may be due to increasing acid exposure time in the esophageal mucosa [36]. Evidence matching NSAID use and GERD development was established in a prospective endoscopy study that found that NSAID use was an independent risk factor for GERD (odds ratio 2.0; CI: 1.3, 3.0; P<0.001) [37]. On the other hand, we found that patients with esophagitis used significantly more PPI than the control group (<0.0001) with tendency for more frequent use among the advanced esophagitis grades (C and D). This higher prevalence might be attributed to the persistent GERD-related symptoms in the esophagitis group that required more PPI therapy. The fact that PPI did not show pure protection of esophagitis further supported the observation of our study that statins insert protective effects.

The novelty of our current study is defining the association between different risk factors in esophagitis degrees, while most studies cited previously assessed GERD, based on patients' symptoms/questionnaires and not endoscopy findings. Our study displayed high strengths and major key points due to the large cohort analysis conducted. One major point is the fact that all patient reports were reviewed by a single senior gastroenterologist thus, obviating interobserver bias with the assessment of esophagitis grades. In addition, our cohort control is positive controls, compared to esophagitis group which allowed us to analyze our early to advance esophagitis patients. This adds to the correlation studying between risk factors and esophagitis. To the best of our knowledge, the aforementioned classification and comparison were not assessed in previous studies.

Our study has several limitations including the retrospective nature and single center study and the symptoms of GERD were not gathered based on validated questionnaires based on pH monitoring studies evaluation for all patients. Moreover, for the control group, GERD diagnosis is uncertain since no pH monitoring data is available. Therefore, we cannot confirm that this group of patients suffers from GERD, and not from functional heartburn. However, this could be an explanation for the absence of EE and for the lesser use of PPIs, which are less effective in functional heartburn than in GERD.

Another limitation of our study is that we included specific groups of patients who underwent gastroscopy who were externally referred by general practitioners and community gastroenterologist to our center to perform the endoscopy due to refractory symptoms, atypical symptoms (such as chest pain), nonresponder to PPI, and the presence of alarm symptoms. On the other hand, the large cohort of patients, although asymmetrically distributed through the study groups, is the main strength of the study.

In conclusion, we found that statins exerted protective effects to the development of esophagitis in GERD patients. This important finding needs to be validated in future randomized prospective studies coupled with pathophysiological examination to better define the protective role of statins in esophagitis development, since it may have an important clinical implication.

## Figures and Tables

**Table 1:** Demographics and baseline characteristics.

**Parameters**	**Control group **	**Group A**	**Group B**	**Group C**	**Group D**
Patients number	2840	818	402	72	16

Mean age (range)	50.2 ±18.9	46.9±17.8	47.9±17.7	53±18.7	65±15.6

Male (%)	46.5	45	54	64	62.5

Body mass index	29.7	29.5	29.5	29.4	28.6

Active smokers (%)	47.2	77.7	75.3	88.8	75

Alcohol consumers (%)	3.7	4	3.4	4.1	18.7

Use of statins (%)	15.8	8.6	6.4	9.7	6.2

Use of NSAIDS (%)	27.1	34.2	32.1	37.5	25

Use of PPI (%)	46	69	68	79	63

Presence of hiatal hernia (%)	28.5	40.9	41.5	45.8	43.7

**Table 2:** Univariate analysis factors affect the presence of esophagitis comparing the control group to A-D groups and comparing initial (groups A and B) with the advanced (B and C) groups.

**Variables**	**Control group **	**Groups A-D**	**P value**	**Groups A+B**	**Group C+D**	**P value**
Active smokers (%)	47.2	77.5	<0.0001	76.9	86.3	0.02

Use of statins (%)	15.8	8	<0.0001	7.9	9	0.3

Use of NSAIDS (%)	27.1	33.6	<0.0001	35.2	33.5	0.3

Use of PPI (%)	46.5	69.5	<0.0001	69	76	0.08

Presence of hiatal hernia (%)	28.5	41.4	<0.0001	41.1	45.4	0.2

Presence of *H*. *pylori* (%)	66.4	70.4	0.02	69.7	80.6	0.06

**Table 3:** Regression analysis of the factor affecting esophagitis development.

**Variables**	**Odds ratio **	**95**%** confidence interval**	**P value**
**Protective factors**			
**Use of statins**			
(i) Control vs. (A-D)	0.463	CI 0.370–0.579	< 0.0001
(ii) Control vs. A	0.497	0.381–0.647	< 0.0001
(iii) Control vs. B	0.366	0.243–0.551	< 0.0001
(iv) Control vs. C	0.036	0.002–0.583	0.01
(v) Control vs. D	0.354	0.046–2.687	0.3

**Promoting factors**			
**Control vs. A-D**			
(i) Use of NSAIDS	1.362	1.183–1.569	<0.0001
(ii) Presence of hiatal hernia	1.779	1.551–2.040	<0.0001
(iii) Presence of *H*. *pylori*	1.811	1.428–2.298	<0.0001

## Data Availability

The data used to support the findings of this study are available from the corresponding author upon request.

## Abbreviations

GERD: Gastroesophageal reflux disease
HMG-CoA: 3-Hydroxy-3-methylglutaryl-coenzyme A
IFN-*γ*: Interferon gamma
NERD: Nonerosive reflux disease
OR: Odds ratio
PEP: Post-ERCP pancreatitis
PPI: Proton pump inhibitor.
